# Extra Virgin Olive Oil Phenol Extracts Exert Hypocholesterolemic Effects through the Modulation of the LDLR Pathway: In Vitro and Cellular Mechanism of Action Elucidation

**DOI:** 10.3390/nu12061723

**Published:** 2020-06-09

**Authors:** Carmen Lammi, Maria Bellumori, Lorenzo Cecchi, Martina Bartolomei, Carlotta Bollati, Maria Lisa Clodoveo, Filomena Corbo, Anna Arnoldi, Nadia Mulinacci

**Affiliations:** 1 Department of Pharmaceutical Sciences, University of Milan, 20133 Milan, Italy; martina.bartolomei@studenti.unimi.it (M.B.); carlotta.bollati@unimi.it (C.B.); anna.arnoldi@unimi.it (A.A.); 2 Department of Neuroscience, Psychology, Drug and Child Health, Pharmaceutical and Nutraceutical Section, University of Florence, 50019 Florence, Italy; maria.bellumori@unifi.it (M.B.); lo.cecchi@unifi.it (L.C.); nadia.mulinacci@unifi.it (N.M.); 3 Interdisciplinar Department of Medicine, University Aldo Moro Bari, 70125 Bari, Italy; marialisa.clodoveo@uniba.it; 4 Department of Pharmacy-Pharmaceutical Sciences, University Aldo Moro Bari, 70125 Bari, Italy; filomena.corbo@uniba.it

**Keywords:** EVOO phenols, HepG2 cells, hypocholesterolemic, PCSK9, LDL receptor

## Abstract

This study was aimed at investigating the hypocholesterolemic effects of extra virgin olive oil (EVOO) phenols and the mechanisms behind the effect. Two phenolic extracts were prepared from EVOO of different cultivars and analyzed using the International Olive Council (IOC) official method for total phenols, a recently validated hydrolytic procedure for total hydroxytyrosol and tyrosol, and ^1^H-NMR analysis in order to assess their secoiridoid profiles. Both of the extracts inhibited in vitro the 3-hydroxy-3-methylglutaryl co-enzyme A reductase (HMGCoAR) activity in a dose-dependent manner. After the treatment of human hepatic HepG2 cells (25 µg/mL), they increased the low-density lipoprotein (LDL) receptor protein levels through the activation of the sterol regulatory element binding proteins (SREBP)-2 transcription factor, leading to a better ability of HepG2 cells to uptake extracellular LDL molecules with a final hypocholesterolemic effect. Moreover, both of the extracts regulated the intracellular HMGCoAR activity through the increase of its phosphorylation by the activation of AMP-activated protein kinase (AMPK)-pathways. Unlike pravastatin, they did not produce any unfavorable effect on proprotein convertase subtilisin/kexin 9 (PCSK9) protein level. Finally, the fact that extracts with different secoiridoid profiles induce practically the same biological effects suggests that the hydroxytyrosol and tyrosol derivatives may have similar roles in hypocholesterolemic activity.

## 1. Introduction

Cardiovascular disease (CVD) is a leading cause of death worldwide and hypercholesterolemia is one of the main risk factors responsible for the development of this multifactorial disease. The low-density lipoprotein (LDL) fraction transports the majority of plasma cholesterol, and the hepatic LDL receptor (LDLR) is responsible for the cellular LDL-uptake and catabolism [1,2]. In general, the LDLR expression is finely tuned by changes in intracellular cholesterol and a transcription factor, known as the sterol-responsive element binding protein-2 (SREBP-2), plays a critical role in LDLR mRNA expression [3,4]. Among SREBP-2 gene targets, the 3-hydroxy-3-methylglutaryl coenzyme A reductase (HMGCoAR) is particularly important [5]. This enzyme plays a key role in the intracellular cholesterol biosynthesis, since it is the rate controlling enzyme in the mevalonate pathway, which is also regulated by the AMP-activated protein kinase (AMPK) pathway [3]. More in details, the LDLR expression and receptor protein localization at cellular membranes are strictly correlated to the intracellular cholesterol biosynthesis pathway. In fact, the transcription of the LDLR and the genes that are required for cholesterol and fatty acid synthesis are controlled by membrane-bound transcription factors called SREBPs [3], and the intracellular cholesterol acts with a negative feedback inhibition mechanism. The SREBP-2 isoform is responsible for the LDLR and HMGCoAR transcription, and the SREBP-2 maturation is regulated by the intracellular cholesterol homeostasis. In fact, after synthesis, SREBP-2 forms a complex with the SREBP cleavage-activating protein (SCAP) and it is localized in the endoplasmic reticulum (ER) as an inactive precursor (pro-SREBP-2). Sterol deficiency results in the release of SREBP-2/SCAP complex from ER and transport to the Golgi, where pro-SREBP2 is processed further, allowing for it to enter the nucleus and up-regulate transcription of LDLR and HMGCoAR [1,2,3]. The increase of LDLR determines an increased clearance of plasmatic LDL-cholesterol with a reduction of cholesterolemia, one of the main risk factors for CVD progression.

In addition, the activity of the LDLR is regulated by proprotein convertase subtilisin/kexin type 9 (PCSK9) at the post-transcriptional level [6,7]. Briefly, PCSK9 and LDLR both contain functional SREs in their promoters that respond to change in intracellular cholesterol levels through the activation of the SREBP pathway. However, since the HNF1-alpha binding site is unique to the PCSK9 promoter and it is not present in the LDLR promoter, the modulation of PCSK9 transcription through HNF1-alpha sequence does not affect LDLR gene expression. Thus, the HNF1-alpha binding site represents a divergent point to disconnect the co-regulation of PCSK9 from LDLR and other SREBP target genes.

Through its functional ability to bind the LDLR, PCSK9 prevents receptor recycling on the hepatocytes surface and promotes its lysosomal degradation, leading to increased LDL-C levels. For this reason, PCSK9 is a promising alternative target for developing new hypocholesterolemic drugs [8]. It is important to underline that statins increase the PCSK9 expression, which dampens effective LDL clearing by promoting LDLR degradation [9], thereby counteracting the therapeutic effects of these drugs.

Extra virgin olive oil (EVOO) is an unrefined oil that is known for its healthy properties, mainly in the area of CVD prevention. Numerous studies have shown that EVOO reduces blood pressure [10], improves the lipid profile by increasing HDL-cholesterol and reducing LDL-cholesterol and triglyceride levels [11,12], reduces oxidative stress, and inhibits human lipoprotein oxidation, which makes LDL less atherogenic [11,13]. These health benefits are often attributed to the high oleic acid content; however, unlike all other vegetable oils, EVOO contains high amounts of peculiar bioactive molecules, including phenolic compounds comprising the group of secoiridoids, as derivatives of oleuropein and ligstroside, as well as smaller amounts of free hydroxytyrosol and tyrosol [14]. 

The first objective of present study was an investigation of the hypocholesterolemic effects of EVOO phenols and the mechanisms behind this effect. As a second objective, the study aimed to find a relationship between the phenolic profiles and observed activities. For this reason, the investigation was conducted on the phenolic extracts that were obtained from two different cultivars. The former was extracted from the EVOO of cultivar Frantoio cultivated in Tuscany (Italy) and was named BUO, whereas the latter was extracted from the EVOO of cultivar Coratina cultivated in Apulia (Italy) and was named OMN. The detailed phenolic profiles of the two extracts were obtained using the International Olive Council (IOC) official method for total phenols, a recently validated hydrolytic procedure for total hydroxytyrosol and tyrosol, and ^1^H-NMR analysis for the main secoiridoid aldehydes. 

The beneficial effects of EVOO phenols may be mediated via a plethora of biochemical pathways and signaling mechanisms that may act either independently or synergistically. For this reason, after the first positive observations, a deeper mechanistic investigation was undertaken in order to find out how EVOO phenols may modulate the activity of the key targets involved in cholesterol metabolism; i.e., LDLR, SREBP-2, HMGCoAR, and PCSK9. To achieve this goal, HepG2 cells were treated with BUO and OMN extracts and their ability to modulate the LDLR pathway was investigated while using a combination of molecular and functional techniques. 

## 2. Materials and Methods 

### 2.1. Chemicals

All of the reagents and solvents employed were from commercial sources. See “Supplementary Materials” for further details on materials and methods.

### 2.2. Selection of the Extra Virgin Olive Oils

The study was conducted on two EVOO extra virgin olive oil samples, which were collected in the 2017 olive oil campaign. The former was produced by Società Agricola Buonamici SrL (Fiesole, Florence, Italy) and was a monocultivar sample from the typical Tuscan cultivar Frantoio (extract name BUO). The latter was from Azienda Agricola Donato Conserva (Modugno, Bari, Italy) and it was from the typical Apulian cultivar Coratina (extract name OMN). The oils were initially analyzed according to the official analytical methods described in Regulation EEC/2568/91 and further amendments and additions [15], for confirming that they belonged to the extra virgin category.

### 2.3. HPLC-DAD Analysis of Phenols from EVOO

Phenolic compounds were analyzed both before and after acid hydrolysis [16]. The phenols extraction was carried out according to the IOC method [17] in the presence of syringic acid as internal standard. The chromatographic analyses were carried out with a HP 1100 system that was provided with a quaternary pump and a DAD detector (Agilent Technologies, Santa Clara, CA, USA). Phenols were separated using a SphereClone ODS (2), 5 μm, 250 × 4.6 mm id column; the elution was obtained by H_2_O (at pH 2.0 by phosphoric acid), acetonitrile and methanol as eluents, while applying the gradient reported in the IOC method (IOC/T.20/Doc No. 29); flow rate, 1 mL/min, injection volume 20 μL. The chromatograms were registered at 280 nm and syringic acid was used as an internal standard for the quantitative analysis, thus expressing the results as mg tyrosol/kg oil.

The hydroalcoholic extracts obtained as described above were treated by the acid hydrolysis method that was previously proposed in order to evaluate the total contents of free and bound tyrosol and hydroxytyrosol [16]. Briefly, 300 μL of the extract were heated at 80 °C for 2 h in the presence of 300 μL of H_2_SO_4_ 1.0 M, and the solution was then diluted with 400 μL of water. The following chromatographic analysis was carried out in a HP1200 liquid chromatograph that was equipped with a DAD detector (Agilent Technologies, Santa Clara, CA, United States) and a reverse phase (RP) C18 column, 150 × 3 mm (5 μm) Gemini (Phenomenex, Torrance, CA, USA); flow rate, 0.4 mL/min. Eluents: H_2_O acidified to pH 3.2 with formic acid (A) and acetonitrile (B). The linear solvent gradient was applied, as follows: solvent A varied 95% to 70% in 5 min., then to 50% in 5 min., then varied to 2% in 5 min., and stayed in this condition for 5 more min.; finally, solvent A came back to 95% in 2 min. The total time of analysis was 22 min., equilibration time, 10 min. The total content of tyrosol was evaluated using a calibration line built using an authentic standard (purity grade 98%), and considering the chromatographic areas at 280 nm. Regarding hydroxytyrosol, its amount was evaluated again while using the calibration line of tyrosol at 280 nm, but applying the following formula for keeping into account that it is overestimated by about 35%: mg OH-tyrosol = mg tyrosol × 0.65 [18]. All data were expressed as mg/kg oil.

### 2.4. Preparation of the Phenolic Extracts for the Biological Testing

Phenolic compounds were extracted from the two EVOO samples according to the following procedure: 50 g of each EVOO sample were exactly weighted and then put in a 500 mL flask together with 150 mL of MeOH:H_2_O 80:20 solution. The mixture was vigorously hand-shaken for 1 min. The extraction was then performed with the aid of an ultrasound bath for 15 min. The obtained mixture was centrifuged at 5000 rpm for 25 min.; the supernatant was recovered and filtered using PVDF type 0.45 µm 13 mm diameter filters with a 60 mL syringe. The obtained solution was defatted twice with 75 mL of hexane and then evaporated under vacuum at room temperature. The dried extract was then dissolved in ethanol up to a total volume of 10 mL. The obtained solution was then split in 10 vials, the solvent evaporated, thus obtaining 10 aliquots of dried extract (each corresponding to 5 mL of EVOO), to be used for the chemical and biological analysis. By this method, each BUO vial contained approximately 10.1 ± 0.16 mg dry weight, whereas each OMN vial 5.6 ± 0.12 mg of dry weight.

### 2.5. Analysis of the Phenolic Extract

The samples that were prepared for the biological testing were submitted to the HPLC-DAD analysis, as indicated above, as well as to ^1^H-NMR analysis using a literature method [19], by using a 400 MHz instrument Advance 400 (Bruker, Bremen, Germany). For the NMR analysis, each defatted phenolic extract, which was obtained from 5 mL of EVOO, was dissolved in 1 mL of CDCl_3_.

### 2.6. Cell Culture Conditions and Treatment

HepG2 cell line was bought from ATCC (HB-8065, ATCC from LGC Standards, Milan, Italy) and cultured following standard protocol. More details are reported in the Supplementary Materials.

BUO and OMN extracts were tested separately. Briefly, each EVOO extract was diluted using DMSO in order to prepare a stock solution (50 mg/mL), which was diluted to reach the final concentration of 25.0 ug/mL in complete growth DMEM. The growth medium of adherent HepG2 cells was discarded and each diluted EVOO extract in complete DMEM was replaced and then incubated for the desirable incubation time based on the experiments. 

### 2.7. MTT Assay

A total of 3 × 10^4^ HepG2 cells/well were seeded in 96-well plates and treated with 25, 50, 100, and 200 μg/mL of BUO and OMN EVOO extracts, or vehicle (H_2_O) in complete growth media for 48 h at 37 °C under 5% CO_2_ atmosphere. Subsequently, the solvent was aspirated and 3-(4,5-dimethylthiazol-2-yl)-2,5-diphenyltetrazolium bromide (MTT) assay was performed following conditions that are detailed reported in Supplementary Materials.

### 2.8. HMGCoAR Activity Assay

The assay buffer, NADPH, substrate solution, and HMGCoAR were provided in the HMGCoAR Assay Kit (Sigma). The experiments were carried out following the manufacturer’s instructions and conditions that were previously optimized at 37 °C [20]. More detailed information is reported in Supplementary Materials.

### 2.9. Western Blot Analysis

The experiments were performed following conditions previously described [21]. In particular, a total of 1.5 × 10^5^ HepG2 cells/well (24-well plate) were treated with 25.0 μg/mL of BUO or OMN EVOO extracts or pravastatin 1.0 µM for 24 h. After each treatment, the cells were processed for western blotting analysis following conditions that were already optimized and described [20]. More details are available on Supplementary Materials.

### 2.10. In-Cell Western (ICW) Assay

Experiments were performed following the previously described conditions by us elsewhere [22]. Briefly, a total of 3 × 10^4^ HepG2 cells/well were seeded in 96-well plate and, the following day, they were treated with 25 μg/mL of BUO and OMN extracts or Pravastatin 1.0 µM in complete growth medium for 24 h. Subsequently, ICW assay was carried out following the protocol that is available on Supplementary Materials.

### 2.11. Ffluorescent LDL Uptake 

Experiments were carried out following condition already described [23]. Briefly, a total of 3 × 10^4^ HepG2 cells/well were seeded in 96-well plates and then kept in complete growth medium for 2 d before treatment. On the third day, LDL-Uptake was carried out following protocol that is detailed described in the Supplementary Materials.

### 2.12. Statistical Analysis

Statistical analyses were carried out by t-student and One-way ANOVA and Graphpad Prism 7, followed by Brown–Forsythe’s test. Values were expressed as means ± S.D.; *p*-values < 0.05, 0.01, 0.001, and 0.0001 were considered to be significant.

## 3. Results

### 3.1. Characterization of The Phenolic Extracts 

The two monocultivar oils were selected as representative of the high quality EVOO category. Their phenolic contents were determined according to the IOC method and after acidic hydrolysis. This latter method recently validated and applied to approximately 100 EVOO samples [16] was used in this study to obtain more details on the oleuropein and ligstroside derivatives of the two extracts. Indeed, the method allowed for measuring the total content of tyrosol and hydroxytyrosol released during the acid hydrolysis from ligstroside and oleuropein derivatives, respectively (EVOO columns in Table 1). The main differences between the two extracts were that BUO was richer in oleuropein derivatives (444.9 µg/g, measured as total hydroxytyrosol after hydrolysis) and OMN was richer in ligstroside derivatives (308.6 µg/g, measured as total tyrosol after hydrolysis). 

The samples for the biological tests were prepared, starting from 50 mL of each oil, and were accurately defatted with n-hexane. Taking the low water stability of most of the extracted phenols into account, each extract was divided in ten aliquots, dried, and stored at −22 °C until use. Specifically, 10.1 mg of phenol extract were obtained from 5 mL of BUO oil, whereas 5.6 mg from 5 mL of OMN oil. These dried extracts were again submitted to HPLC (dry extracts in Table 1) as well as to ^1^H-NMR analysis (Table 2). The total phenolic contents of the two extracts were significantly different, with the OMN extract being the richest one either before (371.1 ± 7.5 µg/mg) or after hydrolysis (426 ± 12.8 µg/mg). These differences, with respect to the original EVOOs (that showed an inverse situation), can be explained by the diverse dry weights of the extracts that were obtained starting from the same volume of oil. 

As concerning the specific secoiridoids content (measured after hydrolysis), hydroxytyrosol was predominant in the BUO extract (208.0 ± 15.6 µg/g dried extract), while tyrosol was more abundant in the OMN one (275.1 ± 2.7 µg/g dried extract). A ^1^H-NMR analysis was performed on both extracts in order to measure specific ratios between the four main secoiridoids of EVOO, namely oleacein, oleuropein aglycone, oleocanthal, and ligstroside aglycone. The specific ratios were calculated (Table 2) by measuring the integral of the duplets corresponding to the aldehyde protons of the selected molecules in the range between 9.20 and 9.60 ppm (i.e., oleacein, 9.23 ppm; oleocanthal, 9.60 ppm; oleuropein aglycone, 9.55 ppm; ligstroside aglycone, 9.53 ppm) [19]. These ratios highlight the significant differences in terms of secoiridoid molecules between the two extracts, which confirm that the oleuropein derivatives were widely prevalent in the BUO sample, in agreement with the results of the HPLC analysis of the hydrolyzed samples (Table 1). 

### 3.2. The Phenolic Extracts Drop In Vitro the HMGCoAR Activity

**Figure 1:**
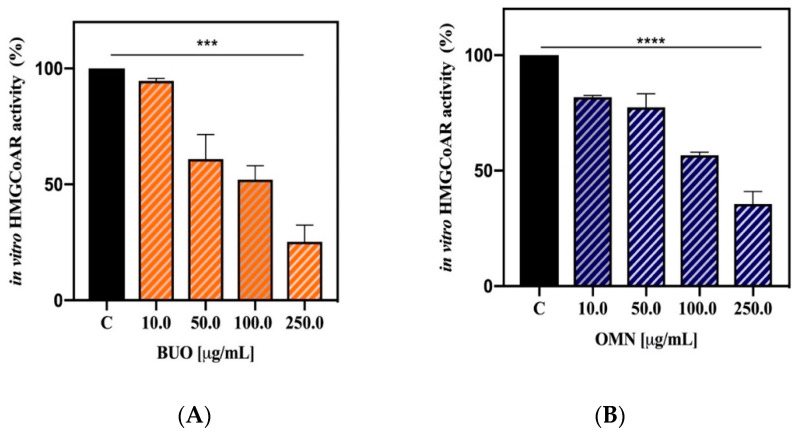
Effect of the phenolic extracts on the modulation of the in vitro HMGCoAR activity. Both BUO (**A**) and OMN (**B**) extracts drop HMGCoAR with dose-response trends. Data represent the mean ± s.d. of six independent experiments performed in triplicate. (***) *p* < 0.001; (****) *p* < 0.0001. C: control sample.

Biochemical investigation was carried out for assessing the phenolic extract capacities to influence the HMGCoAR activity. The results clearly suggest that both extracts inhibit the enzyme activity with dose response trends (Figure 1). In particular, at the concentrations of 10.0, 50.0, 100.0, and 250.0 µg/mL, the BUO extract drops it by 5.4 ± 1.1%, 39.1 ± 10.6%, 48.0 ± 6.0%, and 74.8 ± 7.2%, respectively (Figure 1A), whereas the OMN extract reduces the enzyme activity by 18.2 ± 0.6%, 22.7 ± 6.0%, 43.3 ± 1.4%, and 64.4 ± 5.4%, respectively (Figure 1B).

### 3.3. Effects of the Phenols Extracts on the HepG2 Cell Vitality

**Figure 2:**
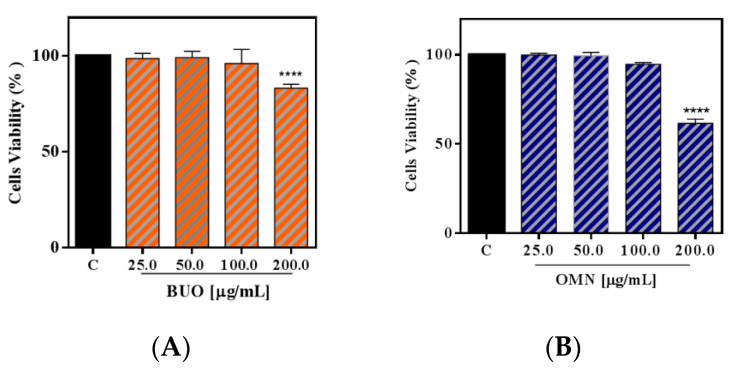
Cell vitality after treatment with phenol extracts. Both BUO (**A**) and OMN (**B**) samples did not affect the HepG2 vitality after 48 h of incubation up to 100 µg/mL. The first cytotoxic effect was observed after the treatment of the hepatic cells with 200 µg/mL (****) *p* < 0.0001. Data represent the mean ± s.d. of three independent experiments performed in triplicate C. untreated HepG2 cells.

Cellular viability experiments were carried out for sorting out those concentrations of the OMN and BUO extract that may potentially produce cytotoxic effects on HepG2 cells. After a 48 h treatment, no cytotoxic effect was observed up to 100 µg/mL versus control cells (C), which suggests that neither BUO nor OMN extracts mediate a cytotoxic effect in this dose range, whereas, at 200 µg/mL, 31.5 ± 2.4% and 16.8 ± 2.7% cell mortalities were observed, respectively (Figure 2A,B). Thus, the following cellular investigations were performed at concentrations that were equal to 25.0 µg/mL.

### 3.4. The Phenol Extracts Modulate the LDLR Pathways

**Figure 3:**
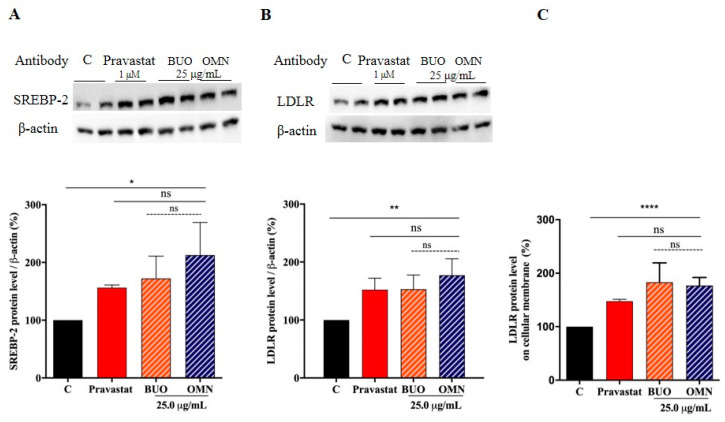
Modulation of the low-density lipoprotein receptor (LDLR) pathway by the phenol extracts. BUO and OMN extracts modulate the intracellular cholesterol pathway through the augmentation of SREBP-2 transcription factor protein level (**A**), which leads to the increase of the LDLR protein levels (**B**). Using In-Cell Western (ICW) assay, the BUON and OMN induction of LDLR localized on the cellular membrane of HepG2 cells is detected (**C**). The experiments were performed using in parallel pravastatin (Pravastat 1.0 µM) as positive control (**A**–**C**). Data represent the mean ± s.d. of eight independent experiments performed in duplicate. (*) *p* < 0.05, (**) *p* < 0.01, (****) *p* < 0.0001. C. untreated HepG2 cells; Pravastat: pravastatin.

Human hepatic HepG2 cells were treated with both extracts at 25.0 μg/mL concentrations to evaluate the ability of the BUO and OMN phenol extracts to modulate the cholesterol metabolism. In parallel, HepG2 cells were treated with pravastatin (1 μM) as reference control. Immunoblotting experiments were carried out on cell lysates. Figure 3A–C clearly indicate that the LDLR pathway is activated after both treatments. More in details, the BUO and OMN extracts up-regulate the protein level of the SREBP-2 transcription factor by 172% ± 38.6% and 212 ± 56.4% (*p* < 0.05), respectively (Figure 3A), and the increase of SREBP-2 protein level lead to an improvement of total LDLR protein levels up to 153 ± 24.4% and 177 ± 28.3% (*p* < 0.001), respectively (Figure 3B). These results are in agreement with the augmentation of LDLR population localized on the hepatocyte surface, which was assessed by an ICW assay. An improvement of the membrane LDLR levels up to 183 ± 36.2% and 177 ± 24.5% (*p* < 0.0001) was observed (Figure 3C). In the same set of experiments, at 1 µM, the positive control pravastatin increased the SREBP-2 by 156 ± 4.6% (*p* < 0.001), the LDLR protein levels by 152 ± 19.7% (*p* < 0.05) (Figure 3A,B), and the membrane LDLR protein level by 50 ± 1.4% (Figure 3C).

### 3.5. The Phenol Extracts Modulate the HMGCoAR Activation by AMPK-Pathway Regulation

**Figure 4:**
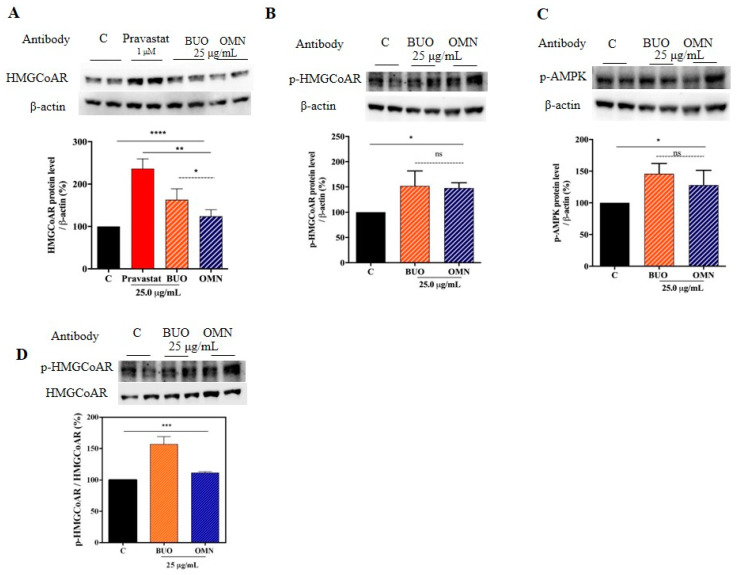
Regulation of the AMPK pathway. Both BUO and OMN samples increase the HMGCoAR protein levels, similarly to pravastatin used as a positive control (**A**). In addition, both extracts induce the activation of AMPK, through the augmentation of its phosphorylation on Thr172 residue, which in turn leads to the increase of the inactive phosphorylated HMGCoAR (p-HMGCoAR) protein levels (**B**–**C**). The ratio between p-HMGCoaR and total HMGCoAR was calculated after treatment with both BUO and OMN samples versus C sample (**D**). Data represent the mean ± s.d. of eight independent experiments performed in duplicate. * (*p)* < 0.05, (**) *p* < 0.01, (***) *p* < 0.001, (****) *p* < 0.0001. C. untreated HepG2 cells.

For evaluating both phenol extract effects on the HMGCoAR protein levels, the HepG2 cells were treated with the extracts (25.0 µg/mL) for 24 h and suitable western blotting experiments were assessed. Upon SREBP-2 transcription factor augmentation, an improvement of the LDLR protein levels was observed as well as an increase of the HMGCoAR protein levels. As shown by Figure 4A, the HMGCoAR protein levels were enhanced by 163 ± 25.6% and 124 ± 15.3%, respectively, while pravastatin (1 μM) increased the enzyme protein levels up to 236.4 ± 23.2% (Figure 4A). Moreover, the treatment with the BUO and OMN extracts significantly increased the phosphorylation levels of HMGCoAR (serine 872, AMPK phosphorylation site) up to 152 ± 29.6% and 148 ± 10.7% (*p* < 0.05), respectively (Figure 4B). This result is in line with the improvement of AMPK phosphorylation (threonine 172) up to 146 ± 16.4% (*p* < 0.05) and 128 ± 23.5% (*p* < 0.05), respectively (Figure 4C).

The pHMGCoAR/total HMGCoAR ratios of treated and untreated cells were also calculated. The ratio of treated cells was higher than that of untreated ones. In fact, the BUO and OMN extracts increased it up to 157 ± 12.2% and 111 ± 2.0%, respectively (*p* < 0.001) (Figure 4D).

### 3.6. The Phenol Extracts Ameliorate HepG2 Ability to Absorb LDL 

**Figure 5:**
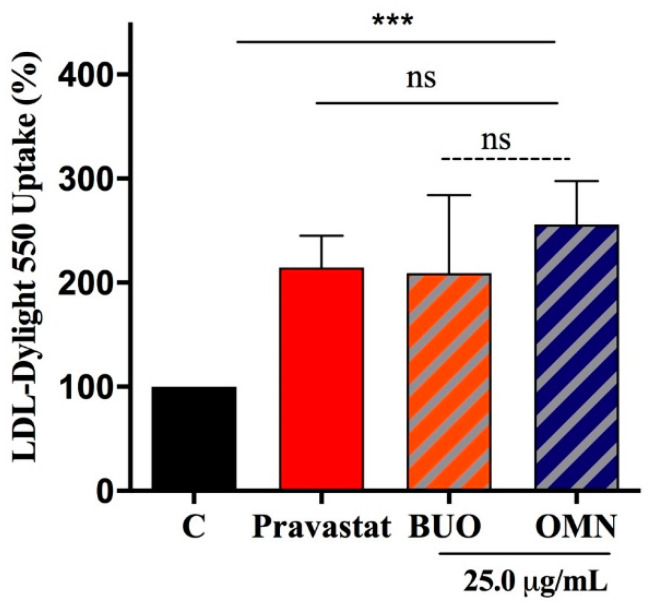
Fluorescent LDL-uptake assay after treatments of HepG2 with the phenol extracts. Both BUO and OMN increase the hepatic cell capability to uptake LDL from the extracellular environment. Experiments were carried out in parallel with pravastatin (Pravastat 1.0 µM) as positive control (B). Data represent the mean ± s.d. of six independent experiments performed in triplicate. (***) *p* < 0.001, ns: not significant. C. untreated HepG2 cells: pravastat: pravastatin.

Functional investigations were performed that aimed to evaluate whether the phenol extracts were able to modulate the HepG2 capacity to uptake extracellular LDL. Indeed, BUO and OMN (25.0 µg/mL) improved the ability of HepG2 cells to absorb LDL-Dylight 550 up to 209 ± 74% and 256 ± 42% (*p* < 0.01), respectively, whereas pravastatin (1 µM) increased it by up to 215 ± 30.8% (*p* < 0.0001) (Figure 5).

### 3.7. The Phenol Extracts Do Not Modulate the Mature PCSK9 Protein Level

**Figure 6:**
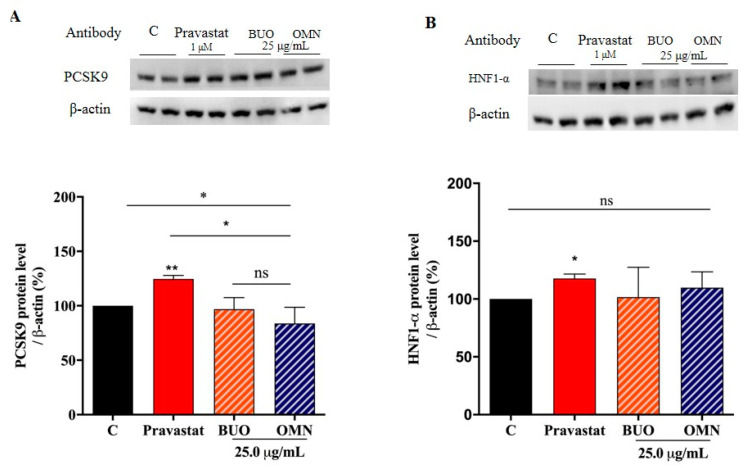
Effects of the phenol extracts on the PCSK9 pathway. BUO and OMN extracts did not affect the PCSK9 protein levels (**A**) and did not produce any effect on the modulation of HNF1-α (**B**). On the contrary, pravastatin (Pravastat 1. µM) increases the PCSK9 protein level (**A**) due to the activation of the HNF1-α a, transcription factor (**B**). Data represent the mean ± s.d. of eight independent experiments performed in duplicate. (*) *p* < 0.05, (**) *p* < 0.01, ns: not significant. C. untreated HepG2 cells; Pravastat: pravastatin.

Both of the extracts were incapable of modulating the mature PCSK9 protein levels and they were also ineffective on the activation of HNF1-α, the PCSK9 transcription factor (Figure 6A,B). On the contrary, pravastatin (1 µM), upon the direct activation of HNF1-α up to 118 ± 3.8% (*p* < 0.05), significantly increased the PCSK9 protein level up to 125 ± 3.4% (*p* < 0.01) (Figure 6A,B).

## 4. Discussion

EVOO is a rich source of phenols that are characterized by antioxidant activity, which counteracts the accumulation of free radicals and, therefore, has a positive effect on human health. Numerous studies have shown the beneficial EVOO effects in improving the lipid profile by increasing HDL-cholesterol and reducing LDL-cholesterol and triglyceride levels, reduces oxidative stress, and inhibits human lipoprotein oxidation, which makes LDL less atherogenic. In addition, the European Food Safety Authority (EFSA) approved the health claim for EVOO phenols, suggesting that their consumption protect LDL particles from oxidative damage.

**Figure 7:**
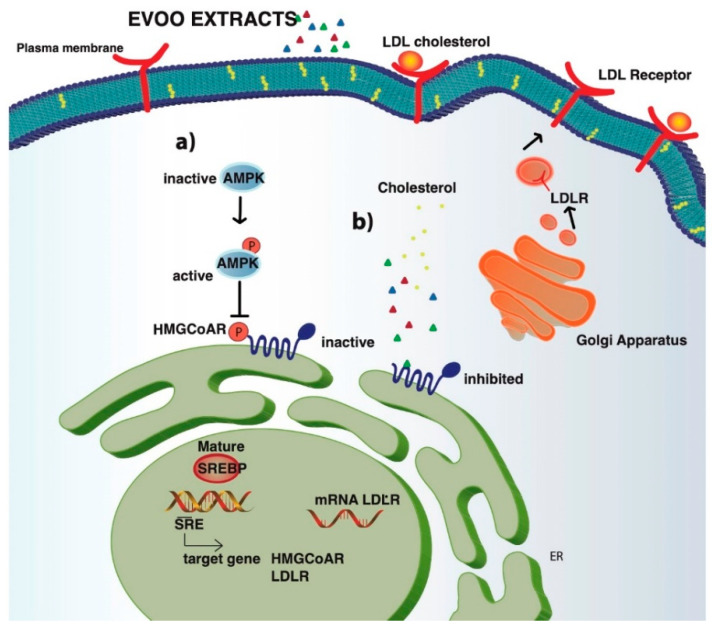
Potential EVOO extracts mechanism of action. Upon cell penetration, EVOO extracts (BUO and OMN) act as competitive inhibitor of HMGCoAR leading to a reduction of intracellular cholesterol synthesis. When intracellular cholesterol level decreases, the transcription factor SREBP2 is activated and LDLR and HMGCoAR genes are transcripted with subsequent increase of LDLR and HMGCoAR protein levels and localization of LDLR in plasma membrane (**a**). In parallel, evidences suggest that in a synergic way, BUO and OMN are able to reduce the cholesterol level production through AMPK-pathway. In particular, both EVOO extracts activate AMPK through an increase of phosphorylation at Thr172, which in turn leads to an inactivation of its target substrate HMGCoAR through phosphorylation at serine 872 (**b**). For this reason, the distinct modulation of the two pathways leads to an increase of the LDLR activity, which can bind and carry the extracellular LDL in HepG2 cells with final hypocholesterolaemic effects.

Based on all these considerations, our study is focused on a deeper mechanistic investigation in order to find out how two EVOO extracts with different phenolic profiles may additionally modulate the cholesterol metabolism, trying to evaluate the role of the secoiridoid compounds, the main phenols in both extracts (Figure 7). 

The results clearly indicate that BUO and OMN extracts are both able to inhibit the HMGCoAR enzyme in a statistically significant way and with a dose dependent manner (Figure 1A,B). These results are in line with a previous study showing that the hepatic HMGCoAR activity is significantly decreased in rats fed a standard diet containing 1% of cholesterol (control diet) enriched with an EVOO phenolic extract versus control rats only fed the control diet [24].

These findings prompted us to investigate in a detailed way the cellular modulation of the LDLR pathway upon HMGCoAR inhibition. The cellular study was carried out on HepG2 cells, because the hepatocyte is the major cell that is involved in the clearance of plasma LDL cholesterol through the LDLR activity. Preliminary MTT experiments were performed in order to exclude any potential cytotoxic effect of both BUO and OMN samples, showing that both EVOO extracts are safe for HepG2 cells up to 100 µg/mL. Based on these results, HepG2 cells were treated with 25.0 µg/mL of each phenol extract, i.e., at a concentration that was about 10-fold lower than the smallest cytotoxic dose and that is a compromise dose between the in vitro HMGCoAR activity assay results and the cytotoxic effects exerted by EVOO extracts on HepG2 cells, respectively. Thus, immunoblotting experiments were assessed in order to investigate the effects on the key targets that are involved in the LDLR pathway. 

Similarly, to 1 µM pravastatin (the positive control), by inhibiting the HMGCoAR activity, these extracts modulate the intracellular cholesterol pathway, leading to an increase of the LDLR and HMGCoAR protein levels through the modulation of SREBP-2 protein level (Figure 3 and Figure 4). The regulation of HMGCoAR is achieved at several levels: transcription, translation, and post-translation (degradation and phosphorylation) levels, respectively [3]. The HMGCoAR transcription and translation increase when the concentrations of the products of the mevalonate pathway are low and decrease when the sterol concentrations are high [25]. The short-term regulation of HMGCoAR is obtained by inhibition through the phosphorylation of Ser872 by AMPK pathway activation. Indeed, the enzyme is physiologically present in the cell in an active non-phosphorylated form (30%) and an inactive phosphorylated one (70%). AMPK is a kinase that plays a key role in cellular energy homeostasis, largely to activate glucose and fatty acid uptake and oxidation when cellular energy is low. Statins are able to activate AMPK, with the consequence of a synergistic inhibition of HMGCoAR activity [26]. In this context, our results show that both phenol extracts induce AMPK activation through its phosphorylation at the Thr172 residue, which, in turn, determines an inhibition of HMGCoAR activity (Figure 4B,C). Indeed, this activation produced an increase of the HMGCoAR phosphorylation levels at Ser872 residue (AMPK phosphorylation site). Therefore, the ratio between pHMGCoAR/total HMGCoAR was calculated for both treated and untreated cells (Figure 4D). When the ratio of treated cells is higher than the ratio of untreated ones, it means that the enzyme is inactivated [27]. Taking all these considerations into account, EVOO phenols extracts are able not only to act as competitive inhibitors of the HMGCoAR in vitro, as suggested by the results that were obtained using in vitro purified catalytic domain of the enzyme, but also to inhibit the cellular HMGCoAR activity by enhancing AMPK activation. As far as the phenolic profiles are concerned, both BUO and OMN extracts are derived from EVOOs that are rich in secoiridoid derivatives of oleuropein and ligstrosides, which may be suggested as being mainly responsible for the observed effects.

Contextually, the modulation of PCSK9 intracellular processing was also investigated. Both SREBP-2 and HNF1-α have been identified as transcriptional activators of PCSK9 gene expression, but only SREBP-2 is able to control the LDLR expression. It is important to observe that statins enhance the level of PCSK9, leading to an increase in the degradation of LDLR, which might be seen as a compensation mechanism that balances the increase of the production of the LDLR [28,29]. In agreement with literature, upon activation of HNF1-α, pravastatin increases the mature PCSK9 protein levels (Figure 6). On the contrary, instead, neither BUO nor OMN extracts (25.0 µg/mL) were able to influence the HNF1-α protein level and, as a direct consequence, they did not modulate the mature PCSK9 protein levels (Figure 6). It is certainly a main outcome of our work to have demonstrated that EVOO phenols are able to ameliorate the cholesterol profile without presenting this relevant drawback of statins. 

The ability of both phenol extracts to positively modulate the LDLR protein levels on the hepatocyte cellular surface was assessed using an ICW assay, i.e., a quantitative colorimetric cell-based assay that allows the detection of target proteins in fixed cultured cells directly in microplates, using a target-specific primary antibody and a horseradish peroxidase-conjugated secondary antibody [22]. In agreement with the ICW results, the treatment with both EVOO phenol extracts improves the ability of HepG2 cells to absorb fluorescent LDL from the extracellular environment, which suggests that this LDLR increase is linked to the functional ability to decrease the cholesterol level from the extracellular environment (Figure 5).

As indicated in the introduction, another objective of the work was to compare the bioactivities of the BUO and OMN extracts whose phenolic compositions are diverse (Table 1 and Table 2). Apparently, the different phenol profile only marginally influences their behaviors, since they share the same mechanism of action at the molecular level. 

In light of these results, it seems possible to affirm that all of the secoiridoid derivatives from the precursors oleuropein and ligstroside are suitable for inducing the same biological effects, which suggests a similar role for the hydroxytyrosol and tyrosol derivatives. This result is partially new considering that only recently it has been confirmed that tyrosol derivatives are more abundant than those of hydroxytyrosol in most EVOOs [16,30], unlike what is indicated in previous literature.

## 5. Conclusions

This study contributes to clarify the mechanism behind the protective effects of EVOO phenols on the cardiovascular system, which suggests a role played not only by hydroxytyrosol, but also by the entire pool of secoiridoid molecules. Indeed, the molecular mechanisms of these EVOO phenol extracts may explain, at least in part, the hypocholesterolemic activity observed so far in many studies that were carried out on the EVOO. 

## Figures and Tables

**Table 1:** Phenolic content in the BUO and OMN samples (extra virgin olive oils (EVOOs) and dry extracts) before and after acid hydrolysis expressed in µg/g in the case of the EVOO and in µg/mg in the case of the dry extracts.

Phenolic Content				
	BUO (before Hydrolysis)		OMN (before Hydrolysis)	
	EVOO (µg/g)	dry extract (µg/mg)	EVOO (µg/g)	dry extract (µg/mg)
Free hydroxytyrosol	9.3 ± 0.9	4.0 ± 0.1	17.4 ± 5.1	16.0 ± 4.6
Free tyrosol	5.1 ± 0.1	2.0 ± 0.1	15.6 ± 5.4	14.0 ± 0.8
Total Phenols	617.9 ± 34.1	289.3 ± 15.6	415.7 ± 7.3	371.1 ± 7.5
	BUO (after Hydrolysis)		OMN (after Hydrolysis)	
	EVOO (µg/g)	dry extract (µg/mg)	EVOO (µg/g)	dry extract (µg/mg)
Total hydroxytyrosol	444.9 ± 33.4	208.0 ± 15.6	169.6 ± 2.6	151.0 ± 3.0
Total tyrosol	332.9 ± 7.9	156.0 ± 3.9	308.6 ± 2.7	275.1 ± 2.7
Tyr + OH-tyr	777.8 ± 41.3	364.1 ± 19.5	478.1 ± 5.3	426.1 ± 5.7

Data are expressed as mean ± SD of three replicates.

**Table 2:** Ratio of integral of signals related to specific monoaldehydic forms of secoiridoids in ^1^H-NMR spectra of the dry extracts from BUO and OMN samples.

Integral Ratio (^1^H-NMR)		
	BUO	OMN
Oleacein/Oleocanthal	1.09	0.49
Oleur A./Ligstr. A	1.73	0.60
Oleacein + Oleur. A/Oleochantal + Ligstr. A	1.4	0.58

Oleur. A, aglycone of oleuropein; Ligstr. A. aglycone of ligstroside.

## Supplementary Materials

The following documents are available online at https://www.mdpi.com/2072-6643/12/6/1723/s1, Brief description of material and methods.

## Abbreviations

**AMPK**	5’ AMP-activated protein kinase
**BSA**	Bovine serum albumin
**CVD**	Cardiovascular disease
**DMEM**	Dulbecco’s modified Eagle’s medium
**EVOO**	extra virgin olive oil
**FBS**	Fetal bovine serum
**HepG2**	Human hepatoma G2
**HMGCoAR**	3-hydroxy-3-methylglutaryl Co-Enzyme A Reductase
**HNF1-α**	Hepatocyte Nuclear Factor 1 alpha
**HPLC-DAD**	high-performance liquid chromatography with a diode-array detector
**HRP**	Horseradish peroxidase
**ICW**	In-cell western
**LDL**	Low density lipoprotein
**LDL-C**	LDL cholesterol
**LDLR**	LDL receptor
**MTT**	3-(4,5-dimethylthiazol-2-yl)-2,5-diphenyltetrazolium bromide
**NMR**	nuclear magnetic resonance
**PBS**	Phosphate buffered saline
**PCSK9**	Proprotein Convertase Subtilisin/Kexin 9
**PMSF**	Phenylmethanesulfonyl fluoride
**RP**	reverse phase
**RT**	Room temperature
**SD**	standard deviation
**SDS**	Sodium Dodecyl Sulphate
**SREBP**	Sterol Regulatory Element Binding Proteins
