# Supplementary material for: Extra Virgin Olive Oil Phenol Extracts Exert Hypocholesterolemic Effects through the Modulation of the LDLR Pathway: In Vitro and Cellular Mechanism of Action Elucidation
Source: Nutrients. 2020 Jun 9;12(6):1723. doi: 10.3390/nu12061723 (PMC7352813; doi:10.3390/nu12061723)
Supplement: Supplementary file 1 [file nutrients-12-01723-s001.pdf]

Supplementary Materials

Extra virgin olive oil phenol extracts exert hypocholesterolemic effects through the modulation of the LDLR pathway: in vitro and cellular mechanism of action elucidation

Carmen Lammi[1*], Maria Bellumori[2], Lorenzo Cecchi[2], Martina Bartolomei[1], Carlotta Bollati[1], Maria Lisa Clodoveo[3], Filomena Corbo[4], Anna Arnoldi[1], Nadia Mulinacci[2].

1. Department of Pharmaceutical Sciences, University of Milan, 20133 Milan, Italy.
2. Department of Neuroscience, Psychology, Drug and Child Health, Pharmaceutical and Nutraceutical Section, University of Florence, 50019 Florence, Italy.
3. Interdisciplinar Department of Medicine University Aldo Moro Bari, 70125 Bari, Italy
4. Department of Pharmacy-Pharmaceutical Sciences, University Aldo Moro Bari, 70125 Bari, Italy

* Correspondence: carmen.lammi@unimi.it; Tel +39-025-031-9372

2. Materials and Methods

2.1. Chemicals

Formic acid, sulfuric acid (95.0-98.0%), methanol and acetonitrile (HPLC grade), ethanol (analytical grade) were from Sigma Aldrich (Steinheim, Germany). Ultrapure water was obtained by the Milli-Q-system (Millipore SA, Molsheim, France). Tyrosol (≥98%) and hydroxytyrosol (≥98%) were purchased from Extrasynthèse (Genay, France). Dulbecco's modified Eagle's medium (DMEM), L-glutamine, fetal bovine serum (FBS), phosphate buffered saline (PBS), penicillin/streptomycin, chemiluminescent reagent, and 24 or 96-well plates were purchased from Euroclone (Milan, Italy). 3-(4,5-Dimethylthiazol-2-yl)-2,5-diphenyltetrazolium bromide (MTT), Janus Green bovine serum albumin (BSA), RIPA buffer, pravastatin, the antibody against β-actin, and HMGCoAR assay kit were bought from Sigma-Aldrich (St. Louis, MO, USA). The antibody against HMGCoAR was bought from Abcam (Cambridge, UK). The antibody against phospho-HMGCoAR (Ser872) was purchased from Bioss Antibodies (Woburn, MA, USA). Phenylmethanesulfonyl fluoride (PMSF), Na-orthovanadate inhibitors, and the antibodies against rabbit Ig-horseradish peroxidase (HRP), mouse Ig-HRP, and SREBP-2 were purchased from Santa Cruz Biotechnology Inc. (Santa Cruz, CA, USA). The antibodies against the LDLR and the phospho- 5' AMP-activated protein kinase (AMPK) (Thr172) were bought from Pierce (Rockford, IL, USA). The antibodies against hepatocyte nuclear factor 1-alpha (HNF1-α) and PCSK9 were bought from GeneTex (Irvine, CA, USA). The inhibitor cocktail Complete Midi from Roche (Basel, Swiss). Mini protean TGX pre-cast gel 7.5%, and Mini nitrocellulose Transfer Packs were purchased from BioRad (Hercules, CA, USA). LDL-DyLight™ 549 from Cayman Chemical (Michigan, USA).

2.2. Selection of the extra virgin olive oils

The study was conducted on two EVOO extra virgin olive oil samples, collected in the 2017 olive oil campaign. The former was produced by Società Agricola Buonamici SrL (Fiesole, Florence, Italy) and was a monocultivar sample from the typical Tuscan cultivar Frantoio (extract name BUO). The latter was from Azienda Agricola Donato Conserva (Modugno, Bari, Italy) and was from the typical Apulian cultivar Coratina (extract name OMN). The oils were initially analyzed according to the official analytical methods described in Regulation EEC/2568/91 and further amendments and additions [15], for confirming that they belonged to the extra virgin category.

2.3. HPLC-DAD analysis of phenols from EVOO

Phenolic compounds were analyzed both before and after acid hydrolysis [16]. The phenols extraction was carried out according to the IOC method [17] in the presence of syringic acid as internal standard. The chromatographic analyses were carried out with a HP 1100 system provided with a quaternary pump and a DAD detector (Agilent Technologies, Santa Clara, CA, USA). Phenols were separated using a SphereClone ODS (2), 5 μm, 250 x 4.6 mm id column; the elution was obtained by H_2O (at pH 2.0 by phosphoric acid), acetonitrile and methanol as eluents, applying the gradient reported in the IOC method (IOC/T.20/Doc No. 29); flow rate, 1 mL/min, injection volume 20 μL. Chromatograms were registered at 280 nm and syringic acid was used as an internal standard for the quantitative analysis, thus expressing the results as mg tyrosol/kg oil.

The hydroalcoholic extracts obtained as described above were treated by the acid hydrolysis method previously proposed in order to evaluate the total contents of free and bound tyrosol and hydroxytyrosol [16]. Briefly, 300 μL of the extract were heated at 80 °C for 2 h in the presence of 300 μL of H_2SO_4 1.0 M, then the solution was diluted with 400 μL of water. The following chromatographic analysis was carried out in a HP1200 liquid chromatograph equipped with a DAD detector (Agilent Technologies, Santa Clara, CA, United States) and a reverse phase (RP) C18 column, 150×3 mm (5 μm) Gemini (Phenomenex, Torrance, CA, USA); flow rate, 0.4 mL/min. Eluents: H_2O acidified to pH 3.2 with formic acid (A) and acetonitrile (B). The linear solvent gradient was applied as follow: solvent A varied 95% to 70% in 5 min, then to 50% in 5 min, then varied to 2% in 5 min and stayed in this condition for 5 more min; finally, solvent A came back to 95% in 2 min. The total time of analysis was 22 min, equilibration time, 10 min. The total content of tyrosol was evaluated using a calibration line built using an authentic standard (purity grade 98%), and considering the chromatographic areas at 280 nm. Regarding hydroxytyrosol, its amount was evaluated again using the calibration line of tyrosol at 280 nm, but applying the following formula for keeping into account that it is overestimated by about 35%: mg OH-tyrosol = mg tyrosol × 0.65 [18]. All data were expressed as mg/kg oil.

2.4. Preparation of the phenolic extracts for the biological testing

Phenolic compounds were extracted from the two EVOO samples according to the following procedure: 50 g of each EVOO sample were exactly weighted and put in a 500 mL flask together with 150 mL of MeOH:H_2O 80:20 solution. The mixture was vigorously hand-shaken for 1 min. The extraction was then performed with the aid of an ultrasound bath for 15 min. The obtained mixture was centrifuged at 5,000 rpm for 25 min; the supernatant was recovered and filtered using PVDF type 0.45 μm 13 mm diameter filters with a 60 mL syringe. The obtained solution was defatted twice with 75 mL of hexane and evaporated under vacuum at room temperature. The dried extract was then dissolved in ethanol up to a total volume of 10 mL. The obtained solution was then split in 10 vials, the solvent evaporated, thus obtaining 10 aliquots of dried extract (each corresponding to 5 mL of EVOO), to be used for the chemical and biological analysis. By this way, each BUO vial contained about 10.1 ± 0.16 mg dry weight, whereas each OMN vial 5.6 ± 0.12 mg of dry weight.

2.5. Analysis of the phenolic extract

The samples prepared for the biological testing were submitted to the HPLC-DAD analysis as indicated above as well as to 1H-NMR analysis using a literature method [19], by using a 400 MHz instrument Advance 400 (Bruker, Bremen, Germany). For the NMR analysis, each defatted phenolic extract, obtained from 5 mL of EVOO, was dissolved in 1 mL of $CDCl_3$.

2.6. Cell culture conditions and treatment

HepG2 cell line was bought from ATCC (HB-8065, ATCC from LGC Standards, Milan, Italy) and was cultured in DMEM high glucose with stable L-glutamine, supplemented with 10% FBS, 100 U/mL penicillin, 100 μg/mL streptomycin (complete growth medium) with incubation at 37 °C under 5% CO_2 atmosphere. HepG2 cells were used for no more than 20 passages after thawing, because a higher number of passages may change the cell characteristics and impair assay results.

BUO and OMN extracts were tested separately. Briefly, each EVOO extract was diluted using DMSO in order to prepare a stock solution (50 mg/mL), which was diluted in order to reach the final concentration of

25.0 ug/mL in complete growth DMEM. The growth medium of adherent HepG2 cells was discarded and each diluted EVOO extract in complete DMEM was replaced and incubated for the desirable incubation time based on the experiments.

2.7. MTT assay

A total of 3 x 10^4 HepG2 cells/well were seeded in 96-well plates and treated with 25, 50, 100, and 200 µg/mL of BUO and OMN EVOO extracts, or vehicle (H_2O) in complete growth media for 48 h at 37 °C under 5% CO_2 atmosphere. Subsequently, the solvent was aspirated and 100 µL/well of 3-(4,5-dimethylthiazol-2-yl)-2,5-diphenyltetrazolium bromide (MTT) filtered solution were added. After 2 h of incubation at 37 °C under 5% CO_2 atmosphere, 0.5 mg/mL solution was aspirated and 100 µL/well of the lysis buffer (8 mM HCl + 0.5% NP-40 in DMSO) added. After 5 min of slow shaking, the absorbance at 575 nm was read on the Synergy H1 fluorescence plate reader (Biotek, Bad Friedrichshall, Germany).

2.8. HMGCoAR activity assay

The assay buffer, NADPH, substrate solution, and HMGCoAR were provided in the HMGCoAR Assay Kit (Sigma). The experiments were carried out following the manufacturer's instructions and conditions previously optimized at 37 °C [20]. In particular, each reaction (200 µL) was prepared adding the reagents in the following order: 1 X assay buffer, pravastatin 1.0 µM or BUO and OMN EVOO extracts (10.0 - 250.0 µg/mL or vehicle (C), the NADPH (4 µL), the substrate solution (12 µL), and finally the HMGCoAR (catalytic domain) (2 µL). Subsequently, the samples were mixed and the absorbance at 340 nm read by the microplate reader Synergy H1 at time 0 and 10 min. The HMGCoAR-dependent oxidation of NADPH and the inhibition properties of samples were measured by absorbance reduction, which is directly proportional to enzyme activity.

2.9. Western blot analysis

Experiments were performed following conditions previously described [21]. In particular, a total of 1.5 × 10^5 HepG2 cells/well (24-well plate) were treated with 25.0 µg/mL of BUO or OMN EVOO extracts or pravastatin 1.0 µM for 24 h. After each treatment, cells were scraped in 30 µL ice-cold lysis buffer [RIPA buffer + inhibitor cocktail + 1:100 PMSF + 1:100 Na-orthovanadate] and transferred in an ice-cold microcentrifuge tube. After centrifugation at 13,300g for 15 min at 4 °C, the supernatant was recovered and transferred into a new ice-cold tube. Total proteins were quantified by Bradford method and 50 µg of total proteins loaded on a pre-cast 7.5% Sodium Dodecyl Sulfate - Polyacrylamide (SDS-PAGE) gel at 130 V for 45 min. Subsequently, the gel was pre-equilibrated with 0.04% SDS in H_2O for 15 min at room temperature (RT) and transferred to a nitrocellulose membrane (Mini nitrocellulose Transfer Packs,) using a trans-Blot Turbo at 1.3 A, 25 V for 7 min. Target proteins, on milk or BSA blocked membrane, were detected by primary antibodies as follows: anti-SREBP2, anti-LDLR, anti-HMGCoAR, anti-phospho AMPK (Thr172), anti-phospho HMGCoAR (Ser872), anti-PCSK9, anti-HNF1-α and anti-β-actin. Secondary antibodies conjugated with HRP and a chemiluminescent reagent were used to visualize target proteins and their signal was quantified using the Image Lab Software (BioRad). The internal control β-actin was used to normalize loading variations.

2.10. In-Cell Western (ICW) assay

Experiments were performed following the conditions previously described by us elsewhere [22]. Briefly, a total of 3 x 10^4 HepG2 cells/well were seeded in 96-well plate and, the following day, they were treated with 25 µg/mL of BUO and OMN extracts or Pravastatin 1.0 µM in complete growth medium for 24 h. Subsequently, they were fixed in 4% paraformaldehyde for 20 min at room temperature (RT). Cells were washed 5 times with 100 µL of PBS/well (each wash was for 5 min at RT) and the endogenous peroxides activity quenched adding 3% H_2O_2 for 20 min at RT. Non-specific sites were blocked with 100 µL/well of 5% Bovine Serum Albumin (BSA, Sigma) in PBS for 1.5 h at RT. LDLR primary antibody solution (1:3000 in 5% BSA in PBS, 25 µL/well) was incubated O/N at +4 °C. Subsequently, the primary antibody solution was discarded and each sample was washed 5 times with 100 µL/well of PBS (each wash was for 5 min at RT). Goat anti-rabbit Ig-HRP secondary antibody solution (Santa Cruz) (1:6000 in 5% BSA in PBS, 50 µL/well), was added and incubated 1

h, at RT. The secondary antibody solution was washed 5 times with 100 μL/well of PBS (each wash for 5 min at RT). Fresh prepared TMB Substrate (Pierce, 100 μL/well) was added and the plate was incubated at RT until desired color is developed. The reaction was stopped with 2 M H_2SO_4 and then the absorbance at 450 nm was measured using the microplate reader Synergy H1.

2.11. Assay for the evaluation of the fluorescent LDL uptake by HepG2 cells

Experiments were carried out following condition already described [23]. Briefly, a total of 3×10^4 HepG2 cells/well were seeded in 96-well plates and kept in complete growth medium for 2 d before treatment. On the third day, cells were treated with 25.0 μg/mL of BUO or OMN extracts or pravastatin 1.0 μM or vehicle (H_2O) for 24 h. At the end of the treatment period, the culture medium was replaced with 50.0 μL/well LDL-DyLight™ 550 working solution. The cells were additionally incubated for 2 h at 37 °C and then the culture medium was aspirated and replaced with PBS (100 μL/well). The degree of LDL uptake was measured using the Synergy H1 fluorescent plate reader (excitation and emission wavelengths 540 and 570 nm, respectively).

2.12. Statistical analysis

Statistical analyses were carried out by t-student and One-way ANOVA and Graphpad Prism 7, followed by Brown-Forsythe's test. Values were expressed as means ± S.D.; p-values <0.05, 0.01, 0.001, and 0.0001 were considered to be significant.
